# Microarray Analysis of Microbiota of Gingival Lesions in Noma Patients

**DOI:** 10.1371/journal.pntd.0002453

**Published:** 2013-09-26

**Authors:** Antoine Huyghe, Patrice François, Andrea Mombelli, Manuela Tangomo, Myriam Girard, Denise Baratti-Mayer, Ignacio Bolivar, Didier Pittet, Jacques Schrenzel, the Geneva Study Group on Noma (GESNOMA)

**Affiliations:** 1 Genomic Research Laboratory. Infectious Diseases Service, University of Geneva Hospitals, Geneva, Switzerland; 2 University of Geneva, Department of Plant Biology, Microbiology Unit, Geneva, Switzerland; 3 Department of Periodontology, School of Dental Medicine, University of Geneva, Geneva, Switzerland; 4 Reconstructive and Plastic Surgery, Faculty of Medicine, Geneva, Switzerland; 5 Institutfür Angewandte Immunologie, Geneva, Switzerland; 6 Infection Control Program, Faculty of Medicine, University of Geneva, Geneva, Switzerland; University of Tennessee, United States of America

## Abstract

Noma (*cancrum oris*) is a gangrenous disease of unknown etiology affecting the maxillo-facial region of young children in extremely limited resource countries. In an attempt to better understand the microbiological events occurring during this disease, we used phylogenetic and low-density microarrays targeting the 16S rRNA gene to characterize the gingival flora of acute noma and acute necrotizing gingivitis (ANG) lesions, and compared them to healthy control subjects of the same geographical and social background. Our observations raise doubts about *Fusobacterium necrophorum*, a previously suspected causative agent of noma, as this species was not associated with noma lesions. Various oral pathogens were more abundant in noma lesions, notably *Atopobium* spp., *Prevotella intermedia*, *Peptostreptococcus* spp., *Streptococcus pyogenes* and *Streptococcus anginosus.* On the other hand, pathogens associated with periodontal diseases such as *Aggregatibacter actinomycetemcomitans*, *Capnocytophaga* spp., *Porphyromonas* spp. and *Fusobacteriales* were more abundant in healthy controls. Importantly, the overall loss of bacterial diversity observed in noma samples as well as its homology to that of ANG microbiota supports the hypothesis that ANG might be the immediate step preceding noma.

## Author Summary

Noma, or *cancrum oris*, is a mutilating disease affecting children in extremely limited-resource countries, suffering poor hygiene and chronic malnutrition. This devastating gangrenous disease affects the hard and soft tissues of the face. To date, the origin of the disease is still debated and current hypotheses rely on microbial diseases or on the immunologic status of the host. In an attempt to better understand the etiology of noma, the authors of the study used microarrays to assess the bacterial microbiota of gingival fluids sampled from 413 healthy and diseased children. Results obtained show reduced bacterial diversity and abundance in samples obtained from diseased patients compared to samples obtained from healthy donors, sharing identical social situation. Oral pathogens were found in both conditions but *Fusobacterium necrophorum*, a putative causative agent of noma, was not associated with the disease. On the other hand, no clear bacterial candidate could be identified as the etiological agent of the disease. However, a number of potential pathogens were found at higher abundance in disease patients compared to healthy donors. Finally, this study provides evidence that acute necrotizing gingivitis often evolves to noma, an observation of importance considering the dramatic condition of patients evolving to acute noma.

## Introduction

Noma, also known as c*ancrum oris*, is a gangrenous disease that typically affects soft as well as hard tissues of the maxillo-facial region. The disorder occurs in young children from poor or less developed areas of the world, mainly in sub-Saharan Africa, but also in Latin America [1] and Asia [2], [3]. The high mortality rate associated with the disease during the acute stage (90% lethality when children are not treated) can be associated with starvation, sepsis or aspiration pneumonia [4]. In 1998, the World Health Organization estimated a global yearly incidence of 140,000 cases [2], but the exact prevalence of the disease is not really known due to distance of villages from medical centers in numerous countries affected by noma. Although the exact etiology of noma remains unknown, several risk factors are thought to play a role in the emergence of the disease [5], [6], such as malaria, measles, tuberculosis, malnutrition or poor oral hygiene. In children living in these less developed countries, acute necrotizing gingivitis (ANG) is suspected to be a precursor stage of noma [7]–[9], but only a small proportion of patients suffering ANG will actually evolve to noma. Falkler [10] suggested that multiple factors such as malnutrition, weakened immune functions and prior viral infections (measles, *Herpesviridae*), all worsened by poor oral hygiene, could play in unison to reduce host resistance and promote the development of oral ulcers. These lesions would then serve as entry sites for the microorganism(s) responsible for the disease. Potential bacterial candidates include *Fusobacterium necrophorum* and *Prevotella intermedia* [10]–[13].

An understanding of the role of specific microorganisms or consortia of multiple organisms in the pathogenesis of noma remains incomplete due to fundamental shortcomings of currently available data. First, when culture techniques are used, the diversity of the captured microbiota inevitably underestimates a large range of fastidious or uncultivable organisms [14]. Second, because the disease develops rapidly and strikes remote geographical areas with poor access to medical facilities, microbiological findings from early cases of noma are sparse. Furthermore, results reported from advanced lesions may reflect changes in local oral ecological conditions enabling or reflecting the development of the disease, rather than identifying its real etiology. Third, this disease prevails in populations by whom even the normal oral flora remains poorly investigated. It therefore remains to be determined if seemingly unusual microbiological findings truly reflect the presence of disease or just geographic location, a particular lifestyle, or a specific socio-economical status.

To improve our knowledge on the etiology of noma, it appears essential to obtain access to early acute cases of the disease, to study in parallel the microbiota of related conditions including healthy controls, and to apply advanced microbiological techniques for studying the diversity of the sampled microbiota. The first step consisted of inventorying the bacterial diversity in oral samples of children in Niger suffering from acute noma or acute necrotizing gingivitis as well as healthy controls by using culture-independent phylogenetic techniques previously described and validated [15]. The specific aim of the next step, reported here, was to validate and extend these observations by developing and using two distinct microarray approaches on an extensive collection of 584 microbial samples obtained during a vast clinical campaign in Niger, where we collected samples in various villages.

## Materials and Methods

### Biological samples

#### Ethics statement

A large epidemiological and sampling campaign was conducted as part of the GESNOMA project from September 2001 to October 2006 in the Zinder area, Niger, Africa. Gingival fluids from children, aged between 6 months and 12 years, affected either by ANG or acute noma were included in the present study. The study complied with the Declaration of Helsinki and was carried out with the prior approval of the Niger Ministry of Health, the WHO noma programme in Niger, and the Swiss non-governmental organization “Sentinelles”. A leaflet (in French) with an explanation of informed consent was translated orally by local staff to the children's parents or guardians. The person responsible for the child (parent or guardian) signed the consent form (usually by a cross and/or a fingerprint). All consent forms were transmitted to the GESNOMA office in Geneva, Switzerland.

The protocol was approved by the Republic of Niger Ministry of Public Health in 2001. The consent forms specifying that fingerprints could be accepted as a signature in the case of illiteracy were approved before use by the Head of the Department of Studies and Programming of the Republic of Niger Ministry of Public Health.

#### Sample selection

In order to establish a clinical diagnosis, every subject underwent general, facial and oral examination with special attention given to the presence of bleeding, gingival pain, ulceration, pseudo-membranes and halitosis. To record clinical and nutritional status, the parents of each child were interviewed by two local healthcare workers of the “Sentinelles” NGO and anthropometric indicator estimates as proposed by the WHO were used [16]. Exclusion criteria were set to select children who had no antibiotic, no dental cleaning and did not receive fortified food during the 3 previous months. Subjects with lesions older than 4 weeks were also excluded. Noma cases were included when they were in acute stage. Acute stage is defined as intraoral lesion (necrotizing stomatitis with exposure of underlining bone), or intraoral lesion with oedema or initial necrosis. Cases with extended necrosis or sequelae of a previous noma were not included. Gingival fluid from the dento-gingival sulcus was collected from both lesion and non-lesion sites of each subject using sterile endodontic paper points, always trying to avoid saliva and blood contamination. For noma cases presenting important destruction of the gingival tissue with no existing sulcus, the sample was collected in the margin of the lesion.

A total of 84 noma subjects and 37 ANG subjects were included in the study, and for each of these subjects both the lesion and non-lesions sites were sampled (Table 1). For healthy controls (n = 343), only one sample was taken from the mandibular anterior tooth. The complete protocol and clinical inclusion criteria were recently reported by our group [17].

**Table 1:** Sample Collection and microarray analysis scheme.

	High-density arrays		Low-density arrays			
	Nb subjects	Nb samples	Nb subjects	Nb samples	Total subjects	Total samples
**ANG**	7	7 LS	30	29 LS	37	36 LS
		7 NLS		30 NLS		37 NLS
**Noma**	19	19 LS	65	65 LS	84	84 LS
		19 NLS		65 NLS		84 NLS
**Controls**	32	32	311	311	343	343
**Total**	**58**	**84**	**406**	**500**	**464**	**584**

LS: Lesion sites ; NLS: Non-lesion sites.

Samples were subsequently stored in guanidinium isothiocyanate medium (RLT buffer, Qiagen) at −80°C until processing.

### High-density phylogenetic microarrays

#### Microarray design and manufacturing

The design of the phylogenetic microarray used for this study was described in Huyghe et al [18]. Briefly, the 25-mer oligonucleotide probes of the array matched the 7 different levels (domain, phylum, class, order, family, genus and species) of the whole bacterial 16S rDNA phylogenetic tree. Probes were selected from the release 9.34 of Ribosomal Database Project [19] (RDP; 194,696 small-subunit rRNA gene sequences) in order to maintain a consistent *Tm* of 60±5°C. The final probe set, composed of 9,477 probes, covered 78.3% of the RDP sequences. To minimize steric hindrance during hybridization, all 25-mer probes were poly(T)-tailed to reach an overall length of 60 nucleotides. Microarrays were manufactured by *in situ* synthesis (Agilent Technologies, Palo Alto, CA).

#### RNA extraction and quantification

Bacterial lysis was performed using 100 mg of glass beads (diameter: 100 µm; Schieritz & Hauenstein, Switzerland) added to the samples. Volume was adjusted to 350 µl with RLT buffer (Qiagen) and samples were vortexed for 1 minute. Total RNA was isolated and purified using the RNeasy Micro Kit (Qiagen) following manufacturer's instructions. Samples were lyophilized and dissolved in 5 µl sterile water. Total RNA quality was assessed using RNA Pico chips on a BioAnalyzer 2100 (Agilent). 16S rRNA levels were assessed by one-step RT-qPCR using 0.2 µM of primers forward: 5′-GGCAAGCGTTATCCGGAATT-3′, reverse: 5′-GTTTCCAATGACCCTCCACG-3′ (Invitrogen, Basel, Switzerland) and 0.1 µM of probe: 5′-CCTACGCGCGCTTTACGCCCA-3′, 5′-end coupled to FAM and 3′-end coupled to TAMRA (Eurogentec, Seraing, Belgium). This assay is designed in a highly conserved region of the bacterial 16S rRNA gene allowing the amplification of most bacterial sequences. One-step RT-qPCR amplification (Invitrogen, final volume of 15 µl) was performed on a SDS 7700 (PE Biosystems, Santa Clara, CA, USA) using the following cycling procedure: t1, 20 min at 50°C; t2, 10 min at 94°C; t3, 15 sec at 94°C; t4, 1 min at 60°C (t3 = t4 were repeated 40 times). Using this strategy, a positive fluorescent signal was consistently obtained between cycles 21–30. Negative samples, typically showing Ct after cycles 33 were considered as degraded and discarded.

#### RNA amplification

The totality of purified RNA was subjected to *in vitro* transcription using the MessageAmp II-Bacteria kit (Ambion, Austin, TX, USA) according to the manufacturer's instructions [20]. Amplified RNA was labeled during *in vitro* transcription in the presence of Cy-3 or Cy-5 cyanine dyes (Perkin-Elmer, Boston, MA, USA). Samples were randomly labeled with Cy-3 or Cy-5 but paired samples originating from the same subject were co-hybridized on the same array. Quality, quantity, amplification efficiency and dye incorporation were evaluated using the NanoDrop ND-1000 Spectrophotometer (NanoDrop Technologies, Inc. Rockland, DE, USA) and the BioAnalyzer 2100 on RNA Nano 6000 chips (Agilent).

#### Microarrays hybridization, scanning and analysis

The bacterial content of 7 ANG and 19 noma subjects was analyzed (see Table 1). For each subject, both lesion and non-lesion site labeled cRNAs were hybridized. For each village, labeled cRNAs from the four healthy control samples were pooled together, for a total number of 32 control samples. Cy5- and Cy3-labelled cRNAs were diluted in a total of 250 µl Agilent hybridization buffer, and hybridized at 60°C for 17 hours in a dedicated hybridization oven (Robbins Scientific, Sunnyvale, CA, USA). Slides were washed, dried under nitrogen flow, and scanned (Agilent) using 100% PMT power for both wavelengths.

Image analysis and signal quantification were achieved using Feature Extraction software (version 6, Agilent). LOWESS (locally weighted linear regression) transformation was used to correct background subtracted signals for unequal dye incorporation. Data were quantile normalized and Log2 transformed. Five groups were analyzed: noma lesion (N, *n* = 19) or non-lesion site (Nn, *n* = 19), gingivitis lesion (G, *n* = 7) or non-lesion site (Gn, *n* = 7), and healthy controls (C, *n* = 32). Statistical significance test consisted in analysis of variance (one-way ANOVA) combined with the Benjamini and Hochberg false discovery rate correction. Differences between groups were considered statistically significant when corrected p-value≤0.05 and fold change ≥2.

Permanova t-statistic and Principal Coordinate Analysis (PCO) were performed on a Bray-Curtis similarity matrix based on hybridization signal intensity in Primer v6 (PRIMER-E Ltd, Plymouth, UK).

### Low-density microarrays

#### Microarray design and manufacturing

Based on the most relevant sequences inventoried by the previously described cloning-sequencing study [15], we designed low-density 16S rDNA arrays. Bolivar and colleagues reported a dataset containing 1,237 partial 16S rRNA gene sequences representing 339 different phylotypes. We used an arbitrary cutoff of 1% of overall abundance to select from this dataset the most abundant sequences for probe design. Using this cutoff, the 132 most abundant 16S rRNA gene sequences were scanned for probes respecting defined physico-chemical properties (*Tm* = 65±5°C; probe length = 23–50 nt; <−5.0 kcal/mol for hairpins; <−8.0 kcal/mol for self-dimers; and dinucleotide repeats shorter than 5 bp) using a commercial software (Array Designer 2.0 by Premier Biosoft). Consequently, the resulting candidate probes were tested for specificity using an improved version of Olicheck [21] with default parameters, leading to a final set of 271 probes. Additionally, probes specific to various Archaea (downloaded from probe Base [22]) and 8 negative control probes (artificial sequences with uniform *Tm*, no secondary structure and containing more than 5 mismatches to known sequences in the public domain) were added to the final probe set. The 335 oligonucleotide probes were synthesized with a C6-linker with free primary amine (Sigma-Aldrich) and spotted on ArrayStrips microarrays (Clondiag GmbH, Jena, Germany).

#### DNA extraction and biotinylation PCR

Extraction of total genomic DNA (gDNA) was performed with glass beads (Sigma-Aldrich, diameter: 212–300 µm) and DNeasy kit (Qiagen) following the manufacturer's protocol. Asymmetrical PCR was chosen in order to favor the amplification of the negative strand. Total gDNA was amplified and biotinylated by duplex PCR using eubacterial universal primers fD1 (0.4 µM) and rD1 (4 µM) as published in [23]. Forward and reverse primers recognized nucleotides 1275–1294 and nucleotides 2759–2795 of the reference sequence J01695 containing the whole *E. coli* ribosomal RNA operon. Each 25 µl reaction contained 15 µl of ReadyMix kit (Sigma-Aldrich, ref. P4600 withMgCl2), 1 nM of biotin-16-dUTP and 10 ng of template DNA. Quality control and quantification of PCR products were assessed by using a NanoDrop ND-1000 Spectrophotometer (NanoDrop Technologies Inc., USA).

#### Microarrays hybridization and scanning

Using agitation, array strips were washed for 5 min with bi-distilled water and 5 min with hybridization buffer at room temperature. Ten µl of labeled amplified DNA was mixed with 90 µl of hybridization buffer, then denatured at 95°C during 2 min and subsequently kept on ice. The denatured sample was incubated in the array strip at 50°C for 1 hour. After hybridization, samples were carefully removed from the array strips, then washed 3 times with proprietary buffers (100 µl Washing Buffer I for 5 min at 30°C, 100 µl Washing Buffer II for 5 min at 20°C and 100 µl Washing Buffer III for 5 min at 20°C). To block the reaction, 100 µl of blocking buffer solution (2% of blocking reagent in 6× SSPE/0.005% Triton ×100) was added in each array and incubated 15 min at 30°C with shaking. Then, the blocking solution was removed and 60 µl of HRP streptavidin (100 pg/µl; Poly-HRP streptavidin by Thermo Scientific in 6× SSPE) was added, and the array strips were incubated for 15 min at 30°C. After removal of the conjugation solution, array strips were washed 3-times during 5 min with proprietary buffers. After removal of the washing solution, 50 µl of staining solution (SeramunGrün by Seramun Diagnostica GmbH) was added and incubated for 5 min at 25°C. After removal of the staining solution, scanning of the microarrays was performed with the Clondiag Array Mate device (Clondiag GmbH, Germany).

#### Data analysis

Signal intensities were extracted using IconoClust software (Clondiag GmbH). For each spot, the extinction signal of local background was subtracted from the intensity value. Normalization and analysis were performed using the Partek Genomic Suite 6.4 (Partek, USA). First, array signals were adjusted in order to normalize the quantity of DNA. Subsequently, each array was normalized against the 8 negative control probes, and then quantile-normalized. Following calibration experiments (not shown), we defined spots with a signal value ≥0.35 and a confidence index ≥0.75 as positive spots. Among the 501 samples hybridized on the low-density arrays and originating from 406 subjects, one ANG sample was discarded from subsequent analysis due to poor hybridization (data not shown). Differences among the 5 conditions (G, *n* = 29; Gn, *n* = 30; N, *n* = 65; Nn, *n* = 65; C, *n* = 311) were sought using ANOVA test, where probes with a fold-change ≥2 and a *P value*≤0.05 were considered as statistically significant.

Permanova and Principal Coordinate Analysis were performed on Bray-Curtis similarity matrix based on hybridization signal intensity in Primer v6 (PRIMER-E Ltd, Plymouth, UK).

## Results

### High-density phylogenetic microarrays

**Figure 1:**
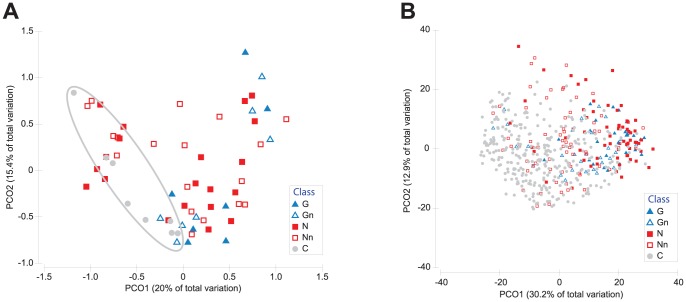
Principal Coordinate Analysis (PCO) plots for the bacterial communities assessed by high-density microarrays specific using probes specific of the genus level (A) and by low-density microarrays (B) using all designed probes. The analysis was based on the Bray-Curtis similarity matrix constructed using normalized signal intensities for the corresponding probes. Gray line delimitates control group samples.

Eighty-four samples were hybridized on the high-density phylogenetic arrays (Table 1).To obtain an overview of the similarity of the bacterial content between all the study subjects we performed a PERMANOVA analysis. The largest differences were found when comparing samples originating from diseased subjects compared to healthy controls (Table 2, p-value between 0.002 and 0.003). To a lesser extent, noma lesion site and non-lesion sites showed significant variations (p = 0.041). However, the comparison of lesion sites sampled from gingivitis and noma subjects showed less variation. The PCO analysis showed some separation between noma, gingivitis and healthy subjects along first two axes, but greater variations were observed in noma samples (Figure 1A). We also did not notice any correlation when taking into account demographic variables of the subjects (data not shown).

**Table 2:** Differences in microbiota profiles among the five groups of samples by PERMANOVA t-statistic with the high-density microarrays.

Groups	P(perm)	t
G vs C	0.002	1.7259
Gn vs C	0.003	1.6373
N vs C	0.002	1.7539
Nn vs C	0.003	1.6745
G vs Nn	0.049	1.3321
N vs Nn	0.041	1.3629
Gn vs N	0.044	1.2932
G vs N	0.074	1.2615
Gn vs Nn	0.271	1.0695
G vs Gn	0.917	0.64808

Statistically significant differences below 0.05 are shown in grey. N: Noma lesion ; Nn: Noma non-lesion ; G: Gingivitis lesion ; Gn: Gingivitis non-lesions ; C: Healthy controls.

**Figure 2:**
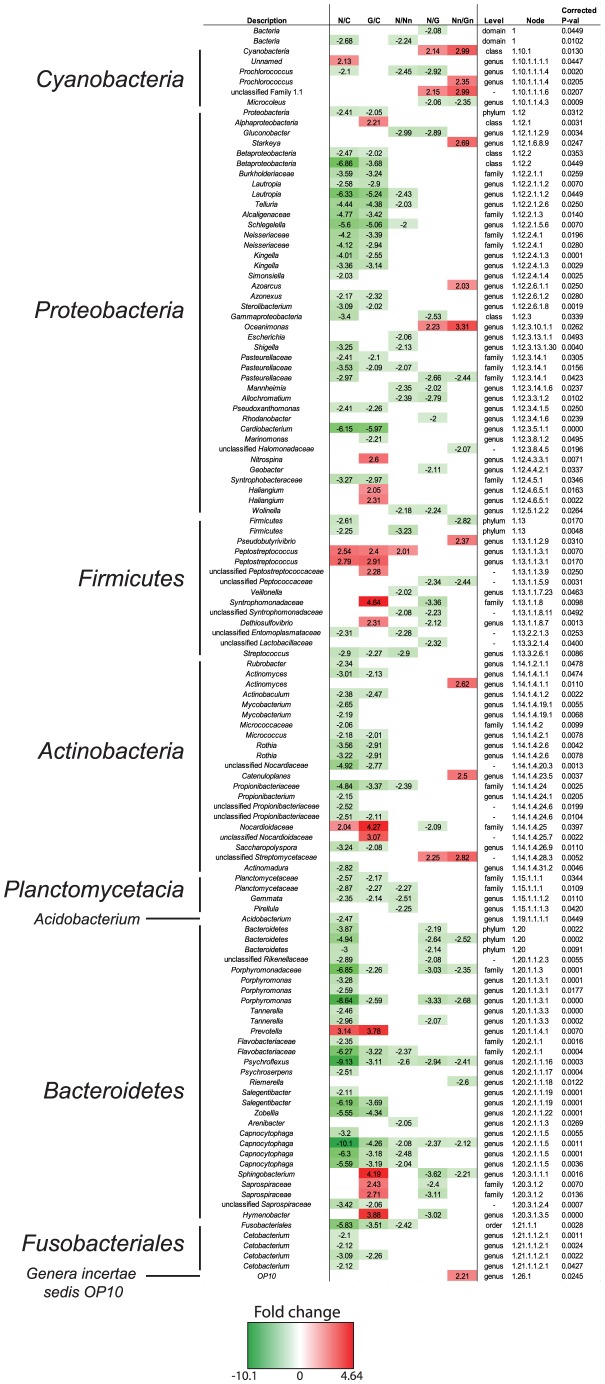
Heatmap of samples hybridized on phylogenetic microarrays. Only probes showing a statistically significant signal between groups (|fold-change| > = 2 and p-value< = 0.05; n = 123) are depicted here. Figures in boxes represent fold-changes. C: Healthy controls; N: Noma lesion site; Nn: Noma non-lesion site; G: Gingivitis lesion site; Gn: Gingivitis non-lesion site.

In order to highlight statistically significant differences, among the probes of the phylogenetic microarray, reflecting imbalance in flora composition across the 5 defined conditions, ANOVA was performed on this dataset. The analysis identified 517 probes showing a p-value≤0.05, of which 123 probes showed a ≥2 fold-change in at least one of the four pair-wise comparisons (Figure 2). The comparison between lesion-sites of noma samples (N) and healthy controls (C) clearly showed the highest number of probes with a significant difference (*n* = 82).

Compared to the healthy controls, a lower bacterial diversity was found in ANG and even a lower one was recorded in noma samples. In both gingivitis and noma lesion-sites, *Peptostreptococcus* spp., *Prevotella* spp. and *Nocardioidaceae* were significantly more represented in lesion sites as compared to their cognate non-lesion sites. On the other side, some taxa are less represented in diseased samples, such as *Cetobacterium* spp., *Rothia* spp. (part of the normal oral flora), *Cardiobacterium* spp. and *Alcaligenaceae.* When comparing diseased sites to healthy controls, the largest difference was found in the groups of the *Firmicutes*, the *Bacteroidetes* and the *Fusobacteriales*. Indeed, less *Porphyromonadaceae*, *Tannerella* spp., *Capnocytophaga* spp. (up to 10-fold changes), *Fusobacteriales* (probe matching Leptotrichia and *Streptobacillus moniliformis*) and *Cetobacterium* spp. (on the basis of the latest RDP release - version 10 - the corresponding probes would be more precisely redefined as matching the *Leptotrichia* genus) were found in noma samples. Other taxa such as *Lautropia* spp. (*Burkholderiaceae* involved in periodontal disease) and *Mycobacterium* spp. were also significantly less represented in noma lesions. However, *Syntrophomonadaceae* and saprophyte phylotypes such as *Nitrospina*, *Haliangium*, *Saprospiraceae*, and *Hymenobacter* appeared over-represented in gingivitis samples only. When comparing diseased sites to healthy controls, one single taxon (described as *Unnamed* in the RDP and included in the *Cyanobacteria* phylum) was found to be more abundant in noma lesions only. This “taxon” includes various environmental organisms such as *Phormidium* spp., *Synechococcus* spp., *Scytonemacrustaceum* and *Calothrixcontarenii*.

Clearly, lesion-sites of gingivitis samples exhibit a higher bacterial diversity than lesion-sites of noma samples (Figure 2: column N/G), notably within the *Bacteroidetes* (including *Porphyromonadaceae* and *Capnocytophaga* spp.) and *Firmicutes* (particularly *Peptococcaceae* and *Syntrophomonadaceae*) phyla. Given that noma lesions stay localized [24], we compared bacterial content of lesion sites to non-lesion sites in an attempt to describe intra-patient differences in microbiota composition (Figure 2: column N/Nn). Noticeably, a broader bacterial diversity is found in non-lesion sites of noma samples, particularly with groups such as *Proteobacteria* (including *Lautropia*, an oral taxon of the *Burkholderiaceae* family), *Bacteroidetes* (including *Capnocytophaga* spp.), *Firmicutes* (notably with *Veillonella* spp. and *Streptococcus* spp.) and *Fusobacteriales*. Conversely, the genus *Peptostreptococcus* is 2-fold more abundant in the lesion sites of our noma samples.

ANG and noma samples, although extremely similar, exhibit some differences, notably in terms of phylotypes classically associated to various environmental milieus: *Cyanobacteria* (including “unclassified Family 1.1”), *Streptomycetaceae* and *Oceanimonas* are more abundant in noma samples. On the contrary, probes matching phylotypes such as *Microcoleus*, *Pasteurellaceae*, *Peptococcaceae*, *Bacteroidetes* (matching essentially *Alistipes* spp. and *Porphyromonas* spp.), *Porphyromonadaceae* (including *Porphyromonas* spp.), *Capnocytophaga*, *Sphingobacterium* and *Psychroflexus* show limited hybridization signals with noma samples.

### Low-density microarray

We designed a 16S rRNA array based on the most relevant sequences inventoried by a cloning sequencing study [15] and obtained from our high-density microarray analysis, in noma, ANG and healthy controls. The purpose of this array is to extrapolate the results obtained by this previous study on a larger scale.

**Figure 3:**
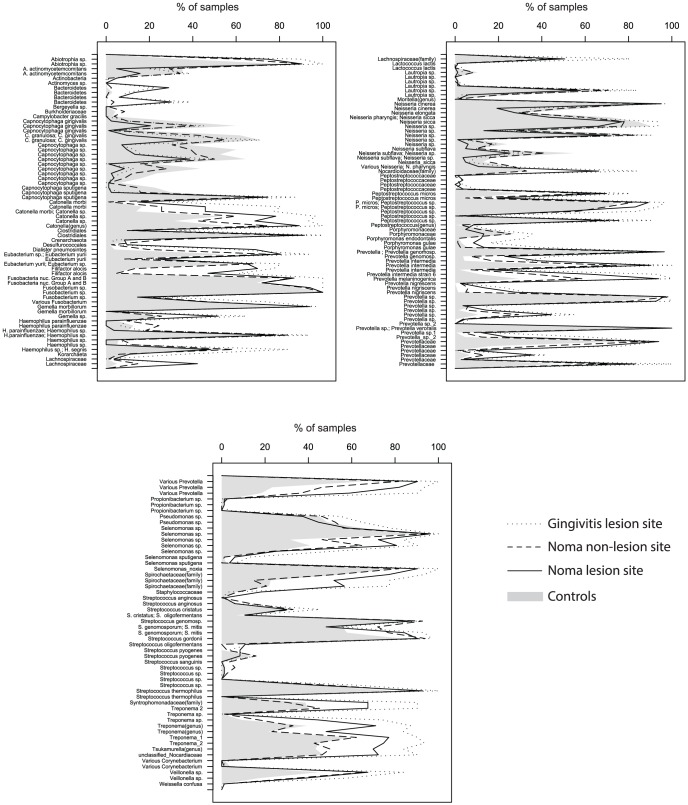
Frequencies of bacteria that are significantly different among subjects.

We successfully labeled and amplified 500 samples, originating from 406 distinct subjects (Table 1) from the five different conditions. ANOVA was performed on our dataset yielding 184 probes with a significant group p-value (P≤0.05). The microbiologic profiles of four groups (noma lesion and non-lesion site, gingivitis lesion site and healthy controls) are depicted in Figure 3. Two genera are present in 100% of all four groups: *Fusobacterium* and *Prevotella*. Overall, some bacteria are found in high abundance in the lesion sites of gingivitis subjects when compared to the other groups. These genera/families include: some *Lachnospiraceae* (including *Catonella* sp. and *Oribacterium* sp.), *Lautropia* sp., *Peptostreptococcus* sp., *Spirochaetaceae* (more particularly *Treponema* sp.) and some *Prevotellaceae*. Conversely, *Capnocytophaga* sp. is found in higher prevalence in healthy samples compared to the other groups. Several genera are found in high abundance in each 4 groups such as: *Abiotrophia* sp., *Clostridiales*, *Fusobacterium* sp., *Gemella morbillorum*, *Neisseria cinerea*, some *Prevotellaceae*, *Streptococcus thermophilus* and *S. gordonii*. Moreover, *P. intermedia* strain 6 is found in higher frequency in lesion sites of ANG and noma (93.5% and 62.6% of the subjects respectively) in comparison to healthy subjects (11.3%); same observation for *P. melaninogenica* with a prevalence of 100% and 94% in the lesion sites of gingivitis and noma respectively, and only 29.3% in healthy subjects. Strikingly, except for some *Lachnospiraceae*, no taxon appears more prevalent in the lesion sites of noma subjects as compared to the 3 other groups.

**Figure 4:**
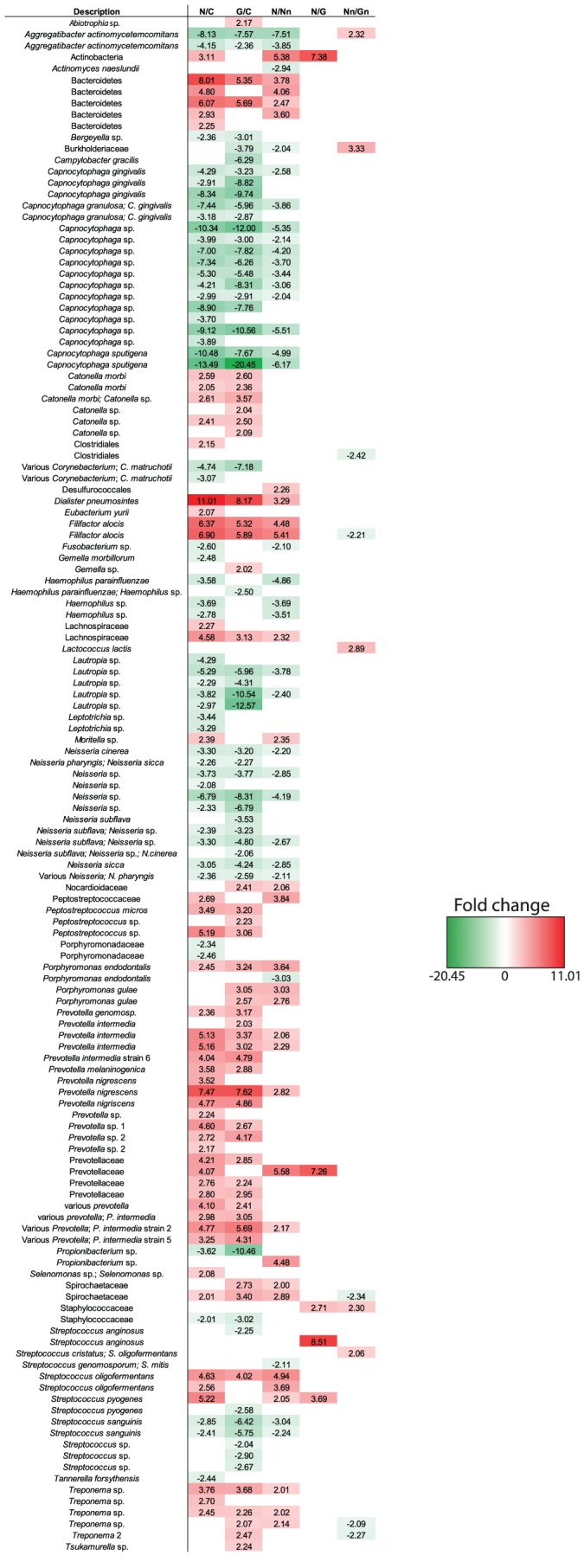
Heatmap of samples hybridized on the low-density microarrays. Figures represent averaged signal intensities fold-changes in the different group comparisons. C: Healthy controls; N: Noma lesion site; Nn: Noma non-lesion site; G: Gingivitis lesion site; Gn: Gingivitis non-lesion site.

ANOVA analysis reveals 135 probes that have statistically significant hybridization rates (P< = 0.05 and |fold-change| ≥2) between gingivitis, noma and healthy groups (Figure 4). Remarkably, three genera and three species are more abundant in healthy sites/subjects when compared to the lesion sites of both noma and gingivitis: *Aggregatibacter actinomycetemcomitans*, *Lautropia* sp., *Neisseria* sp., *Streptococcus sanguinis* and *Capnocytophaga* sp.; this last genus exhibiting the highest fold changes in the control conditions (up to 20.5-fold in gingivitis and 13.5-fold in noma). Conversely, lesion sites present a higher abundance in *Bacteroidetes*, *Dialister pneumosintes* (11-fold in noma), *Filifactor alocis* (*Peptostreptococcaceae*), *Lachnospiraceae*, *Porphyromonas endodontalis*, *Prevotellaceae* (particularly *Prevotella nigrescens* and *P. intermedia*), *Spirochaetaceae* (including *Treponema* sp.) and *Streptococcus oligofermentans*. Note also that the potential pathogens *Leptotrichia* and some *Porphyromonadaceae* are less abundant in noma lesions compared to gingivitis or healthy controls. When compared to gingivitis and controls, lesion sites of noma samples clearly show a higher abundance in *Actinobacteria* (probe matching *Atopobium* spp.), *Prevotellaceae*, *Streptococcus pyogenes*; and *Staphylococcaceae* and *Streptococcus anginosus* when compared to gingivitis only. Interestingly, *Fusobacterium* sp. appears under-represented in the lesion sites of noma samples.

In order to evaluate the relatedness between conditions, we performed a PERMANOVA analysis on the 500 samples (Table 3). The largest variations were found when comparing the lesion sites of either gingivitis or noma to healthy controls (p-value of 0.001), while noma and gingivitis samples showed less, yet significant variations. The dimensional reduction of hybridization profiles by PCO explained 43.1% of the total variance between samples on the two first components (Figure 1B). Although no clear separation between samples is observed, most lesion samples of noma and gingivitis clustered together.

**Table 3:** Differences in microbiota profiles among the five groups of samples by PERMANOVA t-statistic with the low-density microarrays.

Groups	P(perm)	t
G vs Gn	0.001	2.2993
G vs N	0.008	1.5197
G vs Nn	0.001	3.4059
G vs C	0.001	5.2647
Gn vs N	0.001	2.2903
Gn vs Nn	0.011	1.5265
Gn vs C	0.001	2.8101
N vs Nn	0.001	3.4324
N vs C	0.001	6.8325
Nn vs C	0.001	2.9454

Statistically significant differences below 0.05 are shown in grey. N: Noma lesion ; Nn: Noma non-lesion ; G: Gingivitis lesion ; Gn: Gingivitis non-lesions ; C: Healthy controls.

## Discussion

This study is currently the largest effort to evaluate the contribution of microorganisms to the etiology of noma disease. A total of 584 samples were incorporated, representing 464 different subjects and including 84 noma subjects from the region of Zinder in Niger, an endemic noma area. Eighty four samples were hybridized on high density phylogenetic arrays in order to compare global bacterial profiles of our tested populations. In parallel, 500 samples were hybridized on the low-density microarray in an attempt to strengthen the results obtained by Bolivar *et al* [15] on a larger scale. Simultaneously to this work, an epidemiological study aiming at identifying risk factors for noma disease was conducted in the same patient population [17].

Results obtained with both arrays are in agreement and in line with the observations recently published by Bolivar *et al*. [15] using a cloning-sequencing strategy. However, a few taxa were only characterized by either one of the two approaches. Several factors can explain these disparities. First, the low-density arrays were designed in order to detect the most abundant phylotypes (showing a prevalence ≥1%) characterized with the cloning-sequencing strategy described by Bolivar et al. Much like a cloning-sequencing approach, our phylogenetic approach allows for relative quantification (fold-change in respective abundance), meaning that we measure differences between two conditions. Second, the probe set of our phylogenetic array is not comprehensive; 78.3% of the sequences of the RDP (release 9.34) are included, thus, some 16SrDNA sequences cannot be assessed. Finally, cloning sequencing for bacterial identification can suffer from biases inherent to the microbial DNA extraction [25], the PCR technique or the sequences of primer Indeed, the specificity of the so-called “universal” primers can be limited for certain taxa [26]–[28] and 16S rRNA libraries are not necessarily representative of true prokaryotic diversity.

Studies comparing subgingival samples from various forms of periodontal diseases have shown that bacterial microbiota diversity was higher in diseased patient than in healthy ones [29]. In our study, comparison between control samples from healthy donors with that obtained from sick patients (ANG and noma) showed the contrary. In interpreting this divergence one should consider that ANG and noma lesions are fundamentally different from periodontal pockets on the macro- and microscopic levels and may provide different local ecological conditions, hereby influencing the composition of the recovered microbiota. Whereas ANG and noma lesions are fresh acute ulcerations, the periodontal pocket is a physically protected habitat where microorganisms can develop and mature over prolonged periods of time to form a complex biofilm on a hard, non-shedding tooth surface. A decrease in bacterial diversity from ANG compared to noma may reflect the highly acute status of noma, with increased tissue turnover, imposing drastically higher ecological pressure. We observed that putative periodontal pathogens were more abundant in samples from healthy controls compared to diseased conditions.

Known oral pathogens such as *Aggregatibacter actinomycetemcomitans*, *Capnocytophaga*, *Porphyromonas* and *Fusobacteriales* were more abundant in healthy samples compared to diseased conditions. This is not surprising given the fact that the periodontitis associated microbiota is predominantly anaerobic. Samples from controls and gingivitis represent the microbiota of an established dental plaque biofilm that is more likely capable of providing strictly anaerobic conditions than a recently exposed ulcerating soft tissue surface.

One would expect bacterial taxa to play an important role in noma, either as an initiating primary agent, or as a secondary contributory factor, to be detectable in microbial samples from diseased sites. *Fusobacterium necrophorum* is an opportunistic pathogen associated with necrobacillosis in wallabies, a disease similar to noma in humans [30]. In a previous study [10], this species was considered to play a role in the development of the disease. In our study, members of the genus *Fusobacterium* appear neither prevalent nor more abundant in noma lesions. In addition, other representatives of the *Fusobacteriales* order (more precisely *Cetobacterium*, *Leptotrichia* and *Streptobacillus moniliformis*) were significantly more abundant in samples from healthy donors. Our observations support results obtained during previous cloning-sequencing studies performed by Bolivar [15] and Paster [31], and raise doubts on the involvement of the *Fusobacterium* genus as the etiological agent of noma.

In the present study, *Prevotellaceae* and more precisely *Prevotella intermedia* were clearly associated with noma samples. The presence of *P. intermedia* in noma lesions was already documented in previous studies [10], [15], [32], but was not detected in the 4 noma samples processed in the cloning-sequencing study of Paster and colleagues [31]. This pathogen is encountered in adult periodontitis [33], [34] and is frequently isolated in endodontic infections [35]–[37]. It could participate in the etiology of noma by promoting tissue destruction by its ability to degrade lipids and produce proteolytic enzymes [38]. Moreover, *P. intermedia* produces immunoglobulin A1 (IgA1) proteases which could play a critical role in decreasing the oral mucosal immunity [39], hence promoting development of other pathogens in oral lesions. . In noma lesions, we observed a notable abundance of other phylotypes commonly associated with oral infections, such as dental caries (*Atopobium* [40]), periodontitis (*Prevotella* spp. [29] and *Peptostreptococcus* spp. [41]), dentoalveolar infections (*Prevotella* spp. [42]) or palatal abscess (*S. anginosus* [43]).

*P. intermedia* and *Peptostreptococcus* are microorganisms frequently recovered from children with various oral infections characterized by the formation of pus, such as retropharyngeal abscess [44], purulent nasopharyngitis [45], tonsillitis [46], [47], acute suppurative otitis media [48] and acute suppurative thyroiditis [49].

Additionally, *Steptococcus pyogenes* was recovered in noma lesions in significant amounts. This pathogen can cause a variety of infections such as skin infections (impetigo) and throat infections [50]. *S. pyogenes* is also involved in necrotizing fasciitis [51]; and could potentially participate in the development of noma lesions by the release of various virulence factors such as streptolysin, proteases and exotoxins.

In the context of our primary study, it is interesting to note that *P. intermedia* group and *Peptostreptococcus* sp. are also frequently recovered from infections with a clearly non-bacterial initial cause, such as nosocomial sinusitis in mechanically ventilated children [52], infections after trauma [53], post thoracotomy [54], following tracheotomy and intubation [55], infected hemangioma [56], wound infections following spinal fusion [57] and decubitus ulcers [58]. These findings point to the conclusion that high numbers of members of *P. intermedia* group and *Peptostreptococcus* sp. reflect the colonization or secondary infection of a previous lesion. The hypothesis that these microorganisms are not known as mono-infecting agents further supports this finding. The origin of these microorganisms is important to know as they are identified in mixed infections with aerobes distant from the oral and rhino-oto-laryngeal region, such as causative agents of aspiration pneumonia, lung abscess, empyema, or in intra-abdominal, hepatic, splenic and retroperitoneal abscesses [59], or gangrenous appendicitis [60].

An interesting feature of our study is the presence of certain phylotypes never recovered from the oral cavity, such as *Nitrospina*, *Haliangium or Saprospiraceae*. A similar observation was made in a previous cloning-sequencing study by Paster *et al* [31] on noma subjects. These environment-associated taxa likely colonize the host as advanced noma lesions are open and subject to exogenous contamination.

Our study on noma lesions and ANG showed the presence of species commonly recovered in purulent infections, and identified a microbiota that typically colonizes ulcerative oral conditions. Rather than being a classical bacterial infection, our data corroborate the concept of noma showing the characteristics of an opportunistic infection, implicating a change in the equilibrium between bacteria due to a derailment of host defenses or other influences, where microbial changes are quantitative and not only qualitative.

The observed loss of bacterial diversity and appearance of oral pathogens in noma lesions is interesting for the understanding of the disease. Although no bacterial species was identified as the causative agent of noma, our study gives better insight on the bacteriology of noma. Therefore, we cannot confirm that the alteration of the oral microbiota in noma lesions explains the disease by itself, or if our observations reflect a secondary infection. Additional studies are necessary to decipher the etiology of the disease. Particularly, time series sampling and the utilization of high-throughput sequencing capacity will be instrumental to identify noma etiology.

## Supporting Information

Table S1 **Changes in probe intensity between samples in hybridized on high-density microarrays. The statistic used was a two-tailed Student's t-test assuming equal variance.** P-values were adjusted with Benjamini-Hochberg correction (for each taxonomic level individually). Only taxa showing highly significant (P<0.01) changes in at least one pair-wise comparison are presented. The following pairwise comparisons produced no significant changes at any taxonomic level: G vs Gn, Nn vs Gn, N vs G, Gn vs C and G vs C. *, P<0.01; **, P<0.001; ***, P<0.0001. − and +, increase or decrease in average signal intensity, respectively.
(XLSX)

Table S2 **Changes in probe intensity between samples in hybridized on low-density microarrays.** The statistic used was a two-tailed Student's t-test assuming equal variance. P-values were adjusted with Benjamini-Hochberg correction. Only taxa showing highly significant (P<0.01) changes in at least one pair-wise comparison are presented. The following pairwise comparisons produced no significant changes: N vs G and Nn vs G. *, P<0.01; **, P<0.001; ***, P<0.0001. − and +, increase or decrease in average signal intensity, respectively.
(XLSX)
